# Enhancing the Therapeutic Efficacy of KRAS^G12C^ Inhibitors in Lung Adenocarcinoma Cell Models by Cotargeting the MAPK Pathway or HSP90

**DOI:** 10.1155/2021/2721466

**Published:** 2021-11-23

**Authors:** Ying Liu, Lei Wu, Hong Lu, En Wu, Jun Ni, Xiaorong Zhou

**Affiliations:** ^1^Department of Immunology, Nantong University, School of Medicine, Nantong, Jiangsu 226001, China; ^2^Department of Physical Therapy, Shanghai Yangzhi Rehabilitation Hospital, Shanghai 201619, China; ^3^Department of Rehabilitation, The First Affiliated Hospital of Fujian Medical University, Fuzhou, Fujian 350005, China; ^4^Department of Rehabilitation, The Affiliated Hospital of Nantong University, Nantong, Jiangsu 226001, China

## Abstract

**Background:**

KRAS^G12C^ inhibitors have shown promising efficacy in early clinical trials, but drug resistance compromises their long-term benefits. Therefore, it is critical to understand the mechanisms of drug resistance and to design appropriate combinatory treatments to improve efficacy.

**Methods:**

To understand the comprehensive mechanisms of drug resistance, we treated lung cancer cells with KRAS^G12C^ inhibitors for different periods and performed transcriptional profiling and signaling analysis to identify critical factors and pathways that drive drug tolerance and resistance. We also evaluated several drug combinations in vitro and in vivo to identify potentially effective therapeutics.

**Results:**

We found that the feedback activation of multiple receptor tyrosine kinases (RTKs) may have cooperatively induced intrinsic and adaptive resistance to KRAS^G12C^ inhibitors. Notably, continuous KRAS inhibition induced a multidrug-resistant phenotype, implying that upfront combinatory treatment might be required to treat this group of patients. We also demonstrated that concurrently targeting multiple nodes in the RTK/RAS/RAF/MEK/ERK axis improved the efficacy of KRAS^G12C^ inhibitors, mainly by suppressing the reactivation of the mitogen-activated protein kinase (MAPK) pathway. Moreover, the combined use of HSP90 and KRAS^G12C^ inhibitors effectively induced tumor regression in lung adenocarcinoma models in vitro and in vivo.

**Conclusion:**

Together, our findings revealed mechanisms underlying KRAS^G12C^ inhibitors resistance and provided novel candidate combinatory strategies to improve their anticancer activity.

## 1. Introduction

Lung cancer is the leading cause of cancer-related death worldwide [1]. Based on the histopathological presentation, lung cancer is categorized into two major groups: non-small cell lung carcinoma (NSCLC, ∼85%) and small cell lung carcinoma (SCLC, ∼15%). In NSCLC, adenocarcinoma (ADC) and squamous cell carcinoma (SCC) are the two major subtypes, accounting for ∼80% of the cases in total [2]. Despite traditional platinum-based chemotherapy and radiotherapy, the median survival time of NSCLC patients at advanced disease stages was previously dismal, but the situation has been significantly improved by the use of target drugs since the early 2000s and, more recently, by immunotherapy [2–4]. Numerous recurrent genetic alterations, such as *TP53*, *KRAS,* and *EGFR* mutations, have been identified in NSCLC tumors. Some are defined as oncogenic drivers because they can directly cause lung tumorigenesis and, more importantly, are potential therapeutic targets [5]. For instance, *EGFR* mutations, which exist in nearly 50% of Chinese patients with ADC [6], are well defined as lung cancer drivers and are routinely assessed at diagnosis as a selective biomarker for first-line targeted therapy with EGFR inhibitors [7]. In the last decade, numerous studies have demonstrated that targeted drugs, mainly EGFR and ALK inhibitors, are very effective for treating genetically defined lung cancer patients [3]. Target drugs that bind mutated oncogenic proteins and block their activity in tumor cells are more favorable, as they are usually more robust and less toxic due to their broader therapeutic window [8].

Mutations of RAS family genes (*KRAS, NRAS,* and *HRAS*) are frequently observed in various tumors, including lung cancer, pancreatic cancer, and colon cancer [9]. *KRAS* missense mutations, among which the most common subtype is G12 C (∼40%) followed by G12 V (∼20%), are found in approximately 25% of ADCs and mainly affect codons 12, 13, and 61 [10]. The KRAS protein is a GTPase that plays critical roles in many biological processes, such as promoting cell survival and proliferation. The activity of KRAS is dependent on its switching between a GTP-bound active status and a GDP-bound inactive status, and *KRAS* mutations lock the protein in a GTP-bound active status. This phenomenon leads to the sustained activation of the mitogen-activated protein kinase (MAPK) and phosphatidylinositol 3-kinase (PI3K) pathways, two significant pathways downstream of KRAS which are protumorigenic when abnormally activated by mutant KRAS [11].

Although mutant KRAS are authentic therapeutic targets [11], the development of drugs that directly target mutant KRAS achieved a major breakthrough only recently. KRAS-mutant allele-specific inhibitors, such as AMG 510 (sotorasib) and MRTX849 (adagrasib), can target G12C-mutant KRAS and suppress downstream signaling, thus killing tumor cells harboring KRAS^G12C^ mutations [12, 13]. In May 2021, based on the results of phase I/II clinical trials, sotorasib was granted accelerated approval by the US Food and Drug Administration (FDA) for the treatment of KRAS^G12C^-mutant advanced or metastatic NSCLC patients [14]. In these clinical trials, treatment with sotorasib achieved an objective response rate (ORR) of 37.1% in KRAS^G12C^ NSCLC patients and a disease control rate of 86%, demonstrating the clinical efficacy of sotorasib as a monotherapy [14]. Shortly after that, the FDA also granted a breakthrough therapy designation to adagrasib for KRAS^G12C^-mutant NSCLC [15]. Notably, the median progression-free survival of sotorasib-treated patients was 6.8 months, suggesting that the duration of response in some patients is short-lived, probably due to the development of adaptive resistance [14]. Moreover, the efficacy of KRAS^G12C^ inhibitors in colon cancer is low (ORR = 7%), and the reason for this is not very clear [12, 14].

Paired tumor samples from 38 patients, including 27 NSCLC patients who were initially responsive to adagrasib but later acquired resistance, were analyzed for genetic alterations [16]. Seventeen patients (45%) exhibited putative resistance mechanisms, including high-level amplification of *KRAS*^*G12C*^, secondary *KRAS* mutations, activating mutations of *BRAF*, and *MET* amplification [16]. The roles of these mutations need to be verified in a larger cohort; nevertheless, these data suggest that patients develop acquired resistance by both genetic and epigenetic mechanisms. For example, the results of a study on preclinical models suggest that parallel inhibition of the PI3K pathway improves the antitumor activity of KRAS^G12C^ inhibitors in some cell lines exhibiting intrinsic resistance [17]. In addition, cotargeting EGFR, SHP2, mTOR, CDK4/6, and immune checkpoints can enhance the tumor suppressive activity of KRAS^G12C^ inhibitors in preclinical models [13, 17–21]. More recently, epithelial-mesenchymal transition (EMT) was reported to be involved in resistance to KRAS^G12C^ inhibitors [22]. While these findings suggest that tumor cells can develop intrinsic resistance to KRAS^G12C^ inhibitors by various means, the mechanisms of adaptive resistance to KRAS^G12C^ inhibitors remain largely elusive.

In the present study, we examined the dynamic responses of lung cancer cells to KRAS^G12C^ inhibitors and found that multiple receptor tyrosine kinases (RTKs), including ERBB2, ERBB3, and FGFR1, may contribute to drug tolerance and resistance. Moreover, we demonstrated that upfront vertical targeting of the MAPK pathway (RAS/RAF/MEK/ERK) and combinatory treatment with KRAS^G12C^ and HSP90 inhibitors can significantly enhance the therapeutic efficacy in lung adenocarcinoma models.

## 2. Materials and Methods

### 2.1. Cell Culture and Reagents

H358, Calu-1, and H23 cells were obtained from ATCC (Manassas, VA, USA). Cells were cultured in RPMI-1640 medium supplemented with 10% fetal bovine serum (FBS) and 1% penicillin/streptomycin at 37°C in an incubator with 5% CO_2_. Human FGFR1 cDNA (NM_023110.3) was cloned into the empty lentivirus expression vector pSlenti-CMV-Puro (OBiO Technology, Shanghai, China), and the resulting vector was used to generate H358 cells overexpressing FGFR1. Cell proliferation was examined with a CCK-8 assay. All experiments were performed with mycoplasma-free cells. Other reagent information is provided in Table S1.

### 2.2. Transcriptional Profiling

RNA-seq was performed by the Beijing Genomics Institute (BGI, Shenzhen, China) on the BGISEQ platform. Differential expression analysis was performed using the DESeq2 R package, and the genes with adjusted *p* values < 0.05 were designated as differentially expressed genes (DEGs). Gene ontology (GO) enrichment analysis was performed with the clusterProfiler R package. GO terms with adjusted *p* values < 0.05 were considered significantly enriched. Gene set enrichment analysis (GSEA) was performed using online GSEA software (https://www.gsea-msigdb.org). The gene expression data reported in this paper are deposited into the NCBI GEO database under accession number GSE164326.

### 2.3. Xenograft Studies

H358 cells (5 × 10^6^) in 0.2 ml of PBS were injected subcutaneously into the flanks of female nude mice (5–6 weeks old). The mice were monitored every three days for tumor formation. The treatment began when the tumor reached approximately 100 mm^3^ in size. The mice were randomly (*n* ≥ 5 mice per group) assigned to receive AMG 510 by oral gavage (10 mg/kg, daily), STA-9090 by tail vein injection (50 mg/kg, once/week) or a combination; control mice were treated with vehicles. Tumor volume was calculated using the formula (length × width^2^)/2. Studies were performed in compliance with a protocol and institutional guidelines approved by the Ethical Committee of Nantong University.

### 2.4. Real-Time PCR

RNA was extracted using the RNeasy Mini Kit (Qiagen, Germantown, MD), and cDNA was synthesized using the SuperScript VILO cDNA Synthesis Kit (Thermo Fisher, Richardson, TX). Real-time PCR was performed by using the ABI StepOnePlus system (Thermo Fisher) and iTaq™ Universal SYBR Green Supermix (Bio-Rad, Hercules, CA). For data analysis, the 2^−ΔΔCT^ method was used to calculate the fold changes. GAPDH expression was considered to be unaffected under our treatment conditions and was used as a reference gene. The primer sequences used for real-time PCR were as follows (5′-3′): *FGFR1*, forward, CCCGTAGCTCCATATTGGACA, and reverse, TTTGCCATTTTTCAACCAGCG; *GAPDH*, forward, GAAGGTGAAGGTCGGAGTC, and reverse, GAAGATGGTGATGGGATTTC. Each experiment was run in triplicate, and the error bars represent the range of the fold changes calculated from three or four independent experiments.

### 2.5. Immunohistochemistry (IHC)

Serial sections (5 *μ*m) were cut from the tissue blocks, deparaffinized in xylene, and hydrated in a graded series of alcohol. The slides were then immersed in citrate unmasking solution (10X) (CST, Danvers, MA, #14746) in a pressure cooker for 10 minutes for antigen retrieval. After inactivating the endogenous peroxidase activity with 3% H_2_O_2_, the slides were incubated with primary antibodies (Table S1) against Ki67 at a 1 : 100 dilution overnight at 4°C in a humidified chamber. For detection, the slides were treated with the SignalStain Boost Detection system (CST, #8114) according to the manufacturer's instructions, stained with DAB for 3–5 minutes, and counterstained with hematoxylin for 5–15 seconds. Finally, the slides were dehydrated and mounted. All images were obtained using a Zeiss microscope (Observer Z1).

### 2.6. Western Blot

Western blot was performed using whole-cell lysates. Briefly, aliquots of total protein (20–50 *μ*g/lane) were electrophoresed on 10% SDS-polyacrylamide gradient gels and transferred onto polyvinylidene difluoride (PVDF) membranes (Millipore, Bedford, MA, USA). The membranes were incubated at 4°C overnight with primary antibodies against p-ERK, ERK, p-S6, S6, p-FGFR1, FGFR1, p-AKT, AKT, vinculin, and catenin. After rinsing with wash buffer, the membranes were incubated with a horseradish peroxidase-conjugated secondary antibody diluted at 1 : 10,000, and the signal was visualized with SuperSignal West Dura reagents (Thermo Fisher). The antibody information is provided in Table S1.

### 2.7. Statistical Analysis

Statistical analyses were conducted using GraphPad Prism 7 software (GraphPad Software, San Diego, CA, USA). In general, values are plotted as the mean ± standard deviation (SD). Comparisons of means in independent groups were conducted with Student's *t*-test (2 groups) or one-way ANOVA (3 or more groups) for pairwise comparisons, and *p* < 0.05 was considered statistically significant. Other materials and methods are available in the supplemental documents.

## 3. Results

### 3.1. FGFR1 Is Involved in Innate Resistance to KRAS^G12C^ Inhibitors

**Figure 1:**
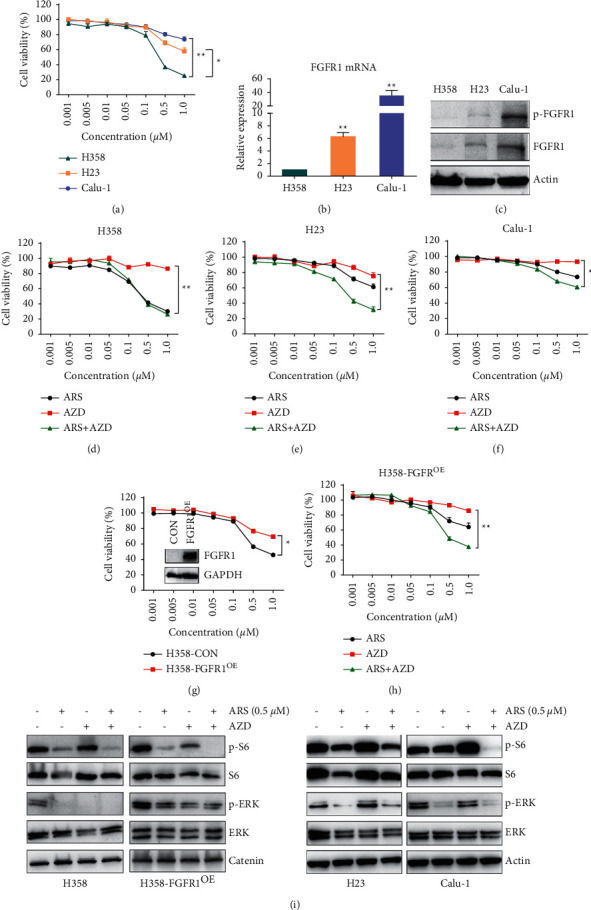
FGFR1 is involved in resistance to KRAS^G12C^ inhibitors in NSCLC cells. (a) CCK-8 assay showing the dose viability responses of NSCLC cell lines treated with ARS-1620 (^*∗*^*p* < 0.05; ^∗∗^*p* < 0.01). (b) Real-time PCR analysis of the FGFR1 levels in H358, H23, and Calu-1 cells. (c) Western blot analysis of FGFR1 and p-FGFR1 in H358, H23, and Calu-1 cells, with *β*-actin serving as a loading control. ((d)-(f)) CCK-8 assays of H358, H23, and Calu-1 cells treated with ARS-1620, AZD4547, or both. (g) Viability of control H358 cells (H358-CON) and H358 cells overexpressing FGFR1 (H358-FGFR1^OE^) as determined by the CCK-8 assay. (h) CCK-8 assays of H358-FGFR1^OE^ cells treated with ARS-1620, AZD4547, or both. (i) Western blot analysis of S6, p-S6, ERK, and p-ERK in H358, H23, Calu-1, and H358-FGFR1^OE^ cells treated with ARS-1620, AZD4547, or both, with catenin or *β*-actin serving as a loading control. All data in the bar graphs of figures are the mean ± SD (*n* ≥ 3). ^*∗*^*p* < 0.05; ^∗∗^*p* < 0.01.

The sensitivity to KRAS^G12C^ inhibitors varies among lung cancer cell lines carrying the KRAS^G12C^ mutation (Figure 1(a)), suggesting the existence of inherent resistance. FGFR1 has been reported to cause resistance to MEK inhibitors in lung cancer cells [23, 24]. We found that the levels of FGFR1 and p-FGFR1 in H358, H23, and Calu-1 cells were correlated with the responses of these cells to ARS-1620 (Figures 1(b) and 1(c)). Combination treatment with ARS-1620 and AZD4547, an FGFR1 inhibitor, for 3 days showed cytotoxic effects on H358 cells that were comparable to those of ARS-1620 treatment alone (Figure 1(d)); however, this treatment exhibited an enhanced tumor cell-killing effect on H23 and Calu-1 cells (Figures 1(e) and 1(f)). We then established H358 cells overexpressing FGFR1 (H358-FGFR1^OE^), and, as expected, forced expression of FGFR1 diminished the cytotoxicity of ARS-1620, whereas combined treatment with ARS-1620 and AZD4547 enhanced the tumor cell-killing effect (Figures 1(g) and 1(h)). Western blot analysis showed that the levels of p-ERK and p-S6 were suppressed by ARS-1620 in all cell lines tested, and adding AZD4547 further reduced the expression of p-S6 (Figure 1(i)), indicating enhanced pathway inhibition. Together, these results demonstrated that the overexpression of FGFR1 in lung cancer cells may contribute to resistance to KRAS^G12C^ inhibitors.

### 3.2. Transcriptional Profiling of KRAS^G12C^ Inhibitor Responses

**Figure 2:**
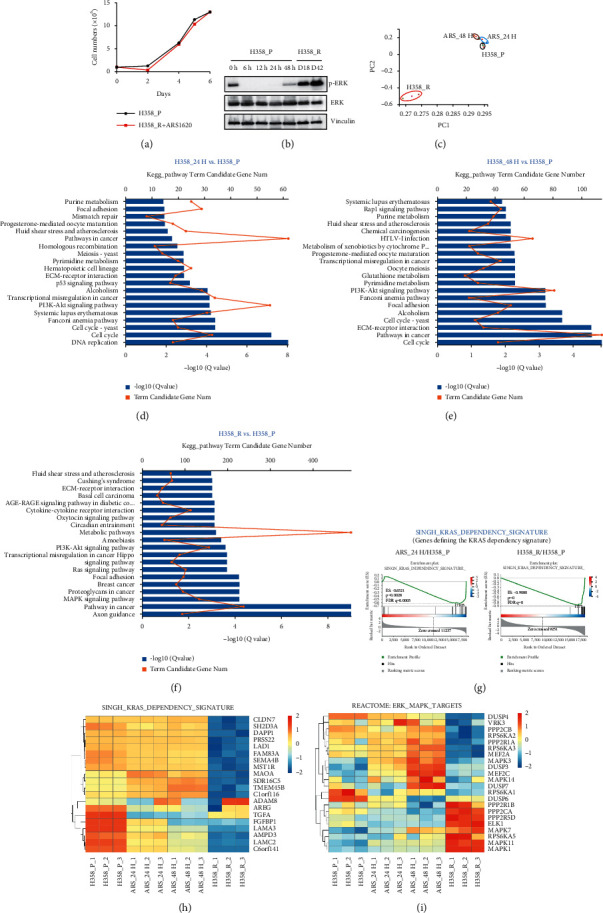
Transcriptional profiling of H358 cells with short- and long-term KRAS^G12C^ inhibition. (a) Growth curves of the parental and resistant H358 cells (H358_P and H358_R). (b) Western blot analysis of p-ERK and ERK in H358_P cells treated for the indicated times and H358_R cells cultured for 18 or 42 days, with vinculin serving as a loading control. (c) Principal component analysis (PCA) of the RNA-seq results. ((d)-(f)) GO enrichment analysis of the DEGs in H358_24H, H358_48H, and H358_R cells compared with H358_P cells. (g) GSEA of the KRAS_DEPENDENCY_SIGNATURE in H358_24H and H358_R cells compared with H358_P cells. ((h)-(i)) Expression heat map of the indicated genes.

In H358 cells, continuous treatment with 1.0 *μ*M ARS-1620 induces acquired resistance, usually within 2–3 weeks. Western blot analysis showed that ARS-1620 inhibited ERK phosphorylation (p-ERK) shortly after administration, but the levels of p-ERK recovered after 48 h. The increases in p-ERK were even more obvious in resistant H358 (H358_R) cells, demonstrating a significant rebound return of ERK activity (Figure 2(b)). To study the dynamic responses to KRAS inhibition, we performed RNA-seq with parental H358 (H358_P) cells and H358_R cells as well as H358 cells treated with ARS-1620 for 24 h (H358_24H) or 48 h (H358_48H). DEGs and significantly altered pathways were identified (Figures 2(c)–2(f), Figure S1A). GSEA and heat map analysis showed that, compared to that in H358_P cells, the KRAS-dependent signature was downregulated in H358_24H and H358_48H cells and more dramatically downregulated in H358_R cells, suggesting that these genes are not essential for the viability of H358_R cells (Figures 2(g) and 2(h)). Consistent with the elevated p-ERK in H358_R cells, GSEA indicated that some of the MAPK and ERK target genes, such as *PPP2R1B, PPP2CA,* and *ELK1*, were downregulated upon short-term treatment but recovered or were overexpressed in H358_R cells (Figure 2(i)), while some genes upregulated by short-term treatment with ARS-1620, such as *MAPK3, DUSP3, and MAPK14*, were suppressed again in the resistant cells (Figure 2(i)).

**Figure 3:**
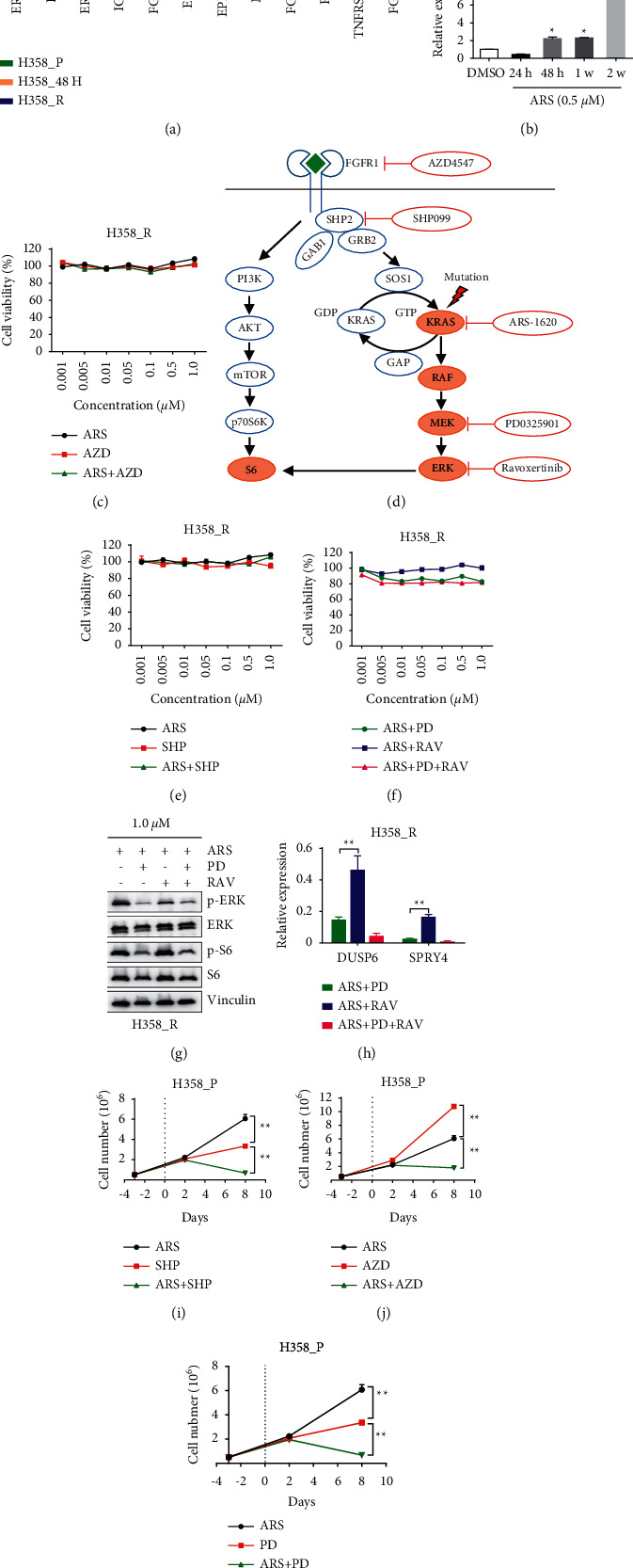
Upfront combinatory treatment enhances the cytotoxicity of ARS-1620. (a) Ratios of the RTK levels in H358_48H and H358_R cells versus H358_P cells. (b) Real-time PCR analysis of the levels of FGFR1 in H358 cells treated with ARS-1620 for the indicated times, with GAPDH serving as an internal control. (c) CCK-8 viability assay for H358_R cells treated with ARS-1620, AZD4547, or both. (d) Schematic diagram of the signaling cascades downstream of FGFR and the drug targets in the MAPK pathway. Mutation of KRAS leads to constitutive activation of the MAPK pathway independent of FGFR1. (e) Viability of H358_R cells upon treatment with the single agent ARS-1620, single agent SHP-099, or the two agents in combination. ((f)-(h)) CCK-8 viability assay of H358_R cells treated with ARS-1620 + PD0325901, ARS-1620 + ravoxertinib, or a three-drug combination. The results of the Western blot analysis of p-S6, S6, p-ERK, and ERK and the real-time PCR analysis of DUSP6 and SPRY4 are shown. ((i)-(k)) CCK-8 viability assay of H358_P cells treated with individual drugs or drug combinations for the indicated periods.

Feedback activation of RTKs causes resistance to targeted therapy in various cancers [25]. Transcriptional profiling revealed distinctive RTK expression patterns in cells treated for short or long periods, as shown in Figure 3(a). Compared to H358_P cells, H358_48H cells showed upregulation of some RTKs, such as *ERBB2/3* and *KDR* (Figure 3(a)), suggesting that they promote cell survival in the acute phase of treatment. Several other RTKs were elevated in H358_R cells, such as *TNFRSF1A* and *FGFR1* (Figure 3(a)), whereas *EGFR*, *MET*, and others were not changed significantly during the entire course of treatment (Figure 3(a)). Real-time PCR indicated that *FGFR1* started to increase at 48 h after treatment with ARS-1620 and was further upregulated at two weeks (Figure 3(b)), the time point at which the drug-tolerant cells began to resume their growth. Together, these data revealed dynamic alterations in multiple signaling pathways and RTK expression in response to KRAS inhibition.

### 3.3. Upfront Combination Treatments Diminish Resistance to KRAS^G12C^ Inhibitors

Surprisingly, H358_R cells were recalcitrant to combined treatment with ARS-1620 and AZD4547 (Figure 3(c)), suggesting that RTKs other than FGFR1 or other pathways support the viability of resistant cells. SHP2 is an adaptor required for the signal transduction of multiple RTKs (Figure 3(d)) [26]; however, cotreatment with ARS-1620 and SHP-099 did not reduce the viability of H358_R cells (Figure 3(e)). Cotreatment with ARS-1620 and PD0325901 (a MEKi) also failed to reduce H358_R cell viability, although MAPK reactivation was prominent in H358_R cells. The cells were even resistant to a three-drug combination of ARS-1620, PD0325901, and ravoxertinib (an ERKi) (Figure 3(f)). Western blot analysis indicated the inhibition of p-ERK and p-S6 (Figure 3(g)), and real-time PCR demonstrated diminished expression of two ERK target genes, *DUSP6* and *SPRY4* (Figure 3(h)), indicating that the lack of effect was not due to suboptimal drug concentrations. Therefore, H358_R cells seemed to display a multidrug-resistant (MDR) phenotype. These results imply that an upfront cotargeting strategy might be more efficacious than a sequential targeting strategy for maximizing the tumor cell-killing effect and preventing the development of adaptive resistance. Indeed, all the drug combinations tested reduced the viability of H358_P cells more effectively than any single-drug treatment, as shown in Figures 3(i)–3(k).

**Figure 4:**
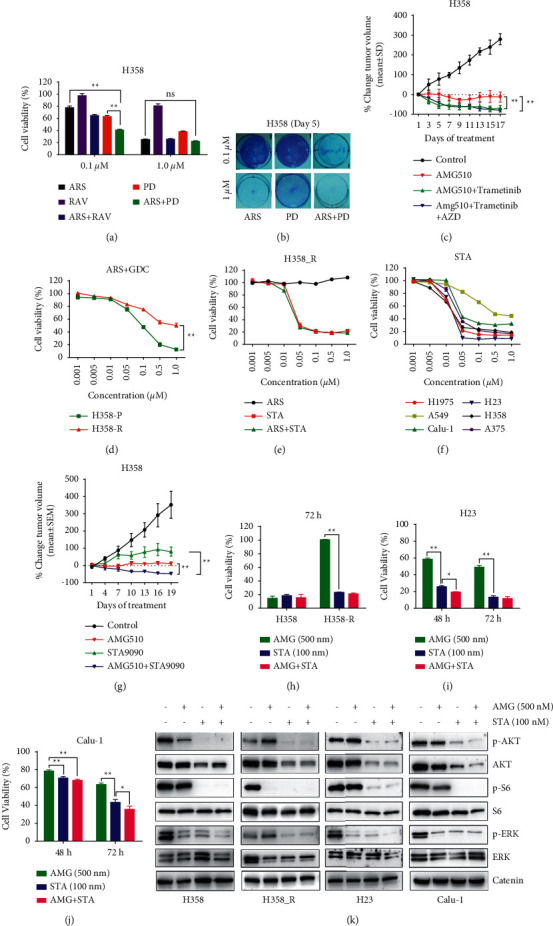
Cotargeting the MAPK pathway and HSP90 enhances the anticancer activity of KRAS^G12C^ inhibitors. (a) CCK-8 viability assay of H358 cells treated with the indicated drug combinations at low or high concentrations. (b) Crystal blue staining of H358 cells treated with ARS-1620 or ARS-1620 in combination with PD0325901 at low or high concentrations. (c) Volumes of H358 xenograft tumors (*n* = 5 for each group) treated with vehicle, AMG 510, AMG 510 + trametinib, or AMG 510 + trametinib + AZD4547. (d) Viability assay of H358_P and H358_R cells treated with ARS-1620 and GDC-094. (e) Viability assay of H358_R cells treated with ARS-1620, STA-9090, or both. (f) Viability analysis of STA-9090 as a single agent in a panel of lung cancer cells and A375 melanoma cells. (g) Volumes of H358 xenograft tumors treated with vehicle, AMG 510, STA-9090, or the two agents in combination (*n* ≥ 5). (h) Viability assay of H358 and H358_R cells treated with AMG 510, STA-9090, or both for 72 h. ((i)-(j)) Viability assay of H23 and Calu-1 cells treated with AMG 510, STA-9090, or both for 48 or 72 h. (k) Western blot analysis of AKT, p-AKT, S6, p-S6, ERK, and p-ERK in H358, H23, Calu-1, and H358_R cells treated with AMG 510, STA-9090, or both for 24 h with catenin serving as a loading control.

Given that the MAPK signaling cascade is activated mainly through the RTK/RAS/RAF/MEK/ERK axis (Figure 3(d)), we hypothesized that cotargeting multiple nodes (vertical targeting) in the pathway would have synergistic effects on signaling inhibition. Indeed, ARS-1620 and PD0325901 in combination at low doses (0.1 *μ*M) were much more potent at killing H358 cells in vitro than any single-drug treatment (Figures 4(a) and 4(b)). Next, H358 cells were transplanted subcutaneously into immunodeficient nude mice, and when the tumors reached 100 mm^3^ in size, the mice were treated with AMG 510 alone or in combination with trametinib. Previous studies have shown that monotherapies with trametinib at 1 mg/kg failed to shrink H358 xenograft sizes [18, 27]. Here, we showed that although AMG 510 monotherapy at 30 mg/kg was not able to significantly shrink H358 tumors, combination therapy with trametinib (1 mg/kg) and AMG 510 effectively induced tumor regression. We also tested the efficacy of AMG 510, trametinib, and AZD4547 (10 mg/kg) in combination, which seemed to induce tumor regression more effectively than the dual-drug combination; however, the difference was not statistically significant (Figure 4(c)). The regimens were well tolerated, as shown by the stable mouse weights over time (Figure S2A). Together, our results suggest that upfront combinatory treatment can improve the efficacy of KRAS inhibitors.

### 3.4. Targeting HSP90 Enhances the Efficacy of KRAS^G12C^ Inhibitors

We showed that H358_R cells no longer respond to treatments that target the downstream MAPK pathway or upstream RTK signaling (Figures 3(c)–3(f)). In addition, when parental H358 cells were treated with ARS-1620 and the PI3K inhibitor GDC-0941, cell viability was dramatically reduced, which is consistent with a previous study [17]. However, cotreatment was less effective in H358_R cells than in H358_P cells (Figure 4(d)), again suggesting that sensitive cells should be initially treated with drug combinations. Interestingly, both H358_P and H358-R cells were susceptible to STA-9090, an HSP90 inhibitor (Figure 4(e)). HSP90 is a chaperone protein involved in a variety of signaling pathways, including the MAPK, PI3K, NF-*κ*B, and JAK-STAT pathways [28], and, not surprisingly, has potent cytotoxic effects on many cell lines harboring MAPK activating events, including H23 and Calu-1 cells (Figure 4(f)).

Next, mice bearing H358 xenografts were treated with AMG 510 (10 mg/kg, daily), STA-9090 (50 mg/kg, once/week), or both for three weeks. The regimens were well tolerated (Figure S2B), and the combined treatment induced tumor regression more effectively than either monotherapy (Figure 4(g)). Consistently, IHC staining of Ki67 in tumor samples showed that tumor cell proliferation was suppressed more significantly by the combination treatment than by AMG 510 monotherapy (Figure S2C).

Treatment with AMG 510 (500 nM), STA-9090 (100 nM), or both drugs in combination for 72 h markedly inhibited H358 cell viability (Figure 4(h)). As expected, H358_R cells were resistant to AMG 510, but their growth was markedly inhibited by STA-9090 alone or by STA-9090 + AMG 510 cotreatment (Figure 4(h)). The growth of H23 and Calu-1 cells was inhibited by AMG 510, although they were less sensitive than H358 cells (Figures 4(i) and 4(j)). STA-9090 also inhibited the growth of H23 and Calu-1 cells, while STA-9090 and AMG 510 in combination inhibited tumor growth better than either drug alone (Figures 4(i) and 4(j)). Next, H358, H358_R, H23, and Calu-1 cells were treated with AMG 510, STA-9090, or the combination for 24 h, and Western blots demonstrated that treatment with AMG 510 alone diminished the levels of p-ERK in H358, H23, and Calu-1 cells; however, it did not affect the levels of p-AKT in H23, Calu-1, and H358_R cells (Figure 4(k)). STA-9090 alone and in combination with AMG 510 dramatically reduced the levels of p-AKT, total AKT, p-S6, and p-ERK in all cell lines tested, and the combination treatment seemed to more effectively reduce the levels of p-ERK (Figure 4(k)). Thus, these results suggested that cotargeting HSP90 is an effective way to enhance tumor killing via KRAS^G12C^ inhibition mainly by inhibiting the activation of the ATK signaling pathway.

## 4. Discussion

The discovery of allele-specific KRAS^G12C^ inhibitors has been a breakthrough, and some pioneering candidates have advanced into early clinical trials and shown encouraging therapeutic efficacy. However, some patients treated with KRAS^G12C^ inhibitors initially achieve tumor regression but relapse later due to the development of drug resistance [12]. Here, we suggest that FGFR1 overexpression contributes to resistance to KRAS^G12C^ inhibitors in lung cancer. Interestingly, FGFR1 also mediates adaptive resistance to MEK inhibitors in *KRAS*-mutant lung cancer cells [24]. Conversely, RAS activation causes resistance to FGFR1 inhibitors in *FGFR1*-amplified lung cancers [29], suggesting that both FGFR1 and KRAS alterations can sustain aberrant MAPK pathway activation and drive reciprocal resistance to targeted drugs in lung cancer therapy (Figure 3(b)).

Manchado et al. reported that *KRAS*-mutant lung cancer cells display either epithelial or mesenchymal phenotypes [17]. In epithelial cells such as H358 cells, feedback upregulation of ERBB3 causes resistance to targeted therapies with MEKi, whereas, in mesenchymal cells such as Calu-1 cells, FGFR1 upregulation is responsible for MEKi resistance [30]. We found ERBB2/3 to be upregulated in H358 cells shortly after ARS-1620 treatment (Figure 2(a)), suggesting that it immediately promotes drug-tolerant cell survival, while mesenchymal transition and FGFR1 overexpression later drive the development of adaptive resistance. Consistently, we found increased expression of the mesenchymal markers VIM, ZEB1, and TWIST1 and decreased expression of the epithelial marker CDH1 in H358_R cells compared to H358_P cells, indicating a mesenchymal phenotype of the resistant cells (Figures S1B–S1E). These results suggest that KRAS inhibition initiates the dynamic expression of multiple RTKs and induces the EMT program in lung cancer cells, culminating in the emergence of a fast-growing resistant population. These results are in line with previous studies suggesting that ZEB1 regulates the expression of FGFR1 in lung cancer cells [30, 31] and that EMT causes resistance to EGFR and BRAF inhibitors [32–34] as well as AMG 510 [22]. Therefore, even in a relatively homogenous cancer cell population, the levels of RTKs dynamically change as the treatment continues. In addition, environmental cues and intertumor and intratumor molecular heterogeneity further complicate the scenario [14]. Hence, targeting multiple RTKs or pathways might be needed to overcome the diverse resistance mechanisms.

Herein, H358_R cells did not respond to the combined treatment with KRASi and FGFRi; however, the treatment was effective in H23, Calu-1, and H358-FGFR1^OE^ cells, all of which showed at least some intrinsic resistance to KRASi treatment alone. Surprisingly, H358_R cells are also resistant to combined treatment with ARS-1620 and SHP2i, although this combination should block the transduction of multiple RTK signaling pathways [26, 35]. Moreover, these cells do not respond to a triple-drug combination that simultaneously targets KRAS^G12C^, MEK, and ERK, although MAPK pathway reactivation is prominent. These phenomena are reminiscent of findings in melanoma patients, as patients who underwent sequential monotherapy became refractory to MEKi monotherapy after relapsing on BRAFi monotherapy. Consequently, upfront combinatory treatment with BRAFi and MEKi has become the first-line therapy in the clinic [36]. We speculate that FGFR1 upregulation, mesenchymal transition, and activation of multiple pathways, including the MAPK and PI3K pathways, collectively induce the multiple therapeutic evasion mechanisms observed in H358_R cells and that upfront combinatory treatment will be clinically favorable, as it may maximize the benefits of targeted therapeutics.

While the mechanisms of resistance are diverse in tumors with activating MAPK events, they are most often associated with the reactivation of the inhibited pathway [37, 38], suggesting that most tumors are addicted to, that is, persistently dependent on, the sustained activation of the MAPK pathway. We propose that the upfront targeting of multiple nodes of the MAPK pathway (vertical targeting) is a reasonable strategy for KRAS^G12C^ lung cancer because it takes advantage of oncogene addiction and thus addresses the primary resistance mechanism. In addition, vertically targeting a signaling chain with drug combinations may more inhibit tumor growth at lower doses of each drug. Indeed, we showed that KRASi and MEKi at low doses effectively killed KRAS^G12C^ tumor cells (Figures 4(a) and 4(b)), in agreement with previous reports showing that combination treatment with RAFi + MEKi or MEKi + ERKi synergistically enhanced tumor killing [39, 40]. Compared with so-called parallel targeting, which may block complementary signals that are critical for normal cell survival, vertical targeting is possibly less toxic [41]. Since AMG 510 and MRTX849 target the mutant KRAS^G12C^ protein specifically, normal cells are thought to be minimally affected. However, clinical trials have shown that approximately 20–30% of lung cancer patients experience side effects greater than grade 3 [12, 14]. Therefore, safety should be carefully evaluated with suitable preclinical models, especially when KRAS^G12C^ inhibitors are combined with other agents.

Targeting HSP90 alone or in combination with KRAS^G12C^ inhibitors effectively killed both parental and resistant cells (Figures 4(e) and 4(h)), likely because HSP90 client proteins are involved in multiple pathways. Thus, targeting HSP90 can overcome resistance via the exertion of broad pharmacological effects [42, 43]. Targeting HSP90 has shown therapeutic potential in *KRAS*-mutant cancer models [44]. HSP90 has also been shown to counteract resistance to targeted therapies or immunotherapies in various cancer models [42, 45, 46]. However, HSP90 inhibitor monotherapy failed to improve the survival of patients with lung cancer, suggesting that drug resistance also limits the efficacy [47]. HSP90 forms a complex with AKT and thus modulates AKT activity [48]. Consistently, we found that STA-9090 decreased the levels of AKT, p-AKT, and p-S6 in lung cancer cells (Figure 4(k)). STA-9090 also decreased the levels of p-ERK in cells with different sensitivities to AMG 510, including resistant H358_R cells (Figure 4(k)). CRAF kinase was reported to be an HSP90 client protein [49], and STA-9090 may thus inhibit ERK activation by blocking the transduction of the RAF/MEK/ERK signaling cascade. Together, these data suggest that targeting HSP90 can simultaneously inhibit multiple pathways that are essential for the survival of lung cancer cells (Figure 3(d)). More importantly, combined treatment with AMG 510 and STA-9090 is tolerable in mice and effectively induces tumor regression better than either drug alone, suggesting that this combination can synergistically kill tumor cells and prevent the emergence of dual-drug-resistant cells.

## 5. Conclusion

Feedback activation of RTKs and multiple pathways and mesenchymal transition may cooperatively promote cell survival and resistance to KRAS^G12C^ inhibitors. Upfront combinatory treatment targeting MAPK-related pathways can improve the efficacy of KRAS^G12C^ inhibitors. Moreover, cotargeting KRAS^G12C^ and HSP90 with the AMG 510 + STA-9090 combination might be an effective therapeutic strategy for lung cancer patients with the KRAS^G12C^ mutation.

## Data Availability

RNA sequencing data are available in the NCBI Gene Expression Omnibus (GEO) database under accession number GSE164326.

## Supplementary Materials

Supplementary Materials Figure S1: transcriptional profiling of H358 cells treated with ARS-1620. Figure S2: the efficacy of combination therapy with AMG 510 and STA-9090 in vivo. Table S1: information on the antibodies and reagents.
