# Supplementary material for: Enhancing the Therapeutic Efficacy of KRASG12C Inhibitors in Lung Adenocarcinoma Cell Models by Cotargeting the MAPK Pathway or HSP90
Source: J Oncol. 2021 Nov 23;2021:2721466. doi: 10.1155/2021/2721466 (PMC8632397; doi:10.1155/2021/2721466)
Supplement: Supplementary Materials — Figure S1: transcriptional profiling of H358 cells treated with ARS-1620. Figure S2: the efficacy of combination therapy with AMG 510 and STA-9090 in vivo. Table S1: information on the antibodies and reagents. [file 2721466.f1.zip › 2721466.f1/Supplemental Figure S1.pdf]

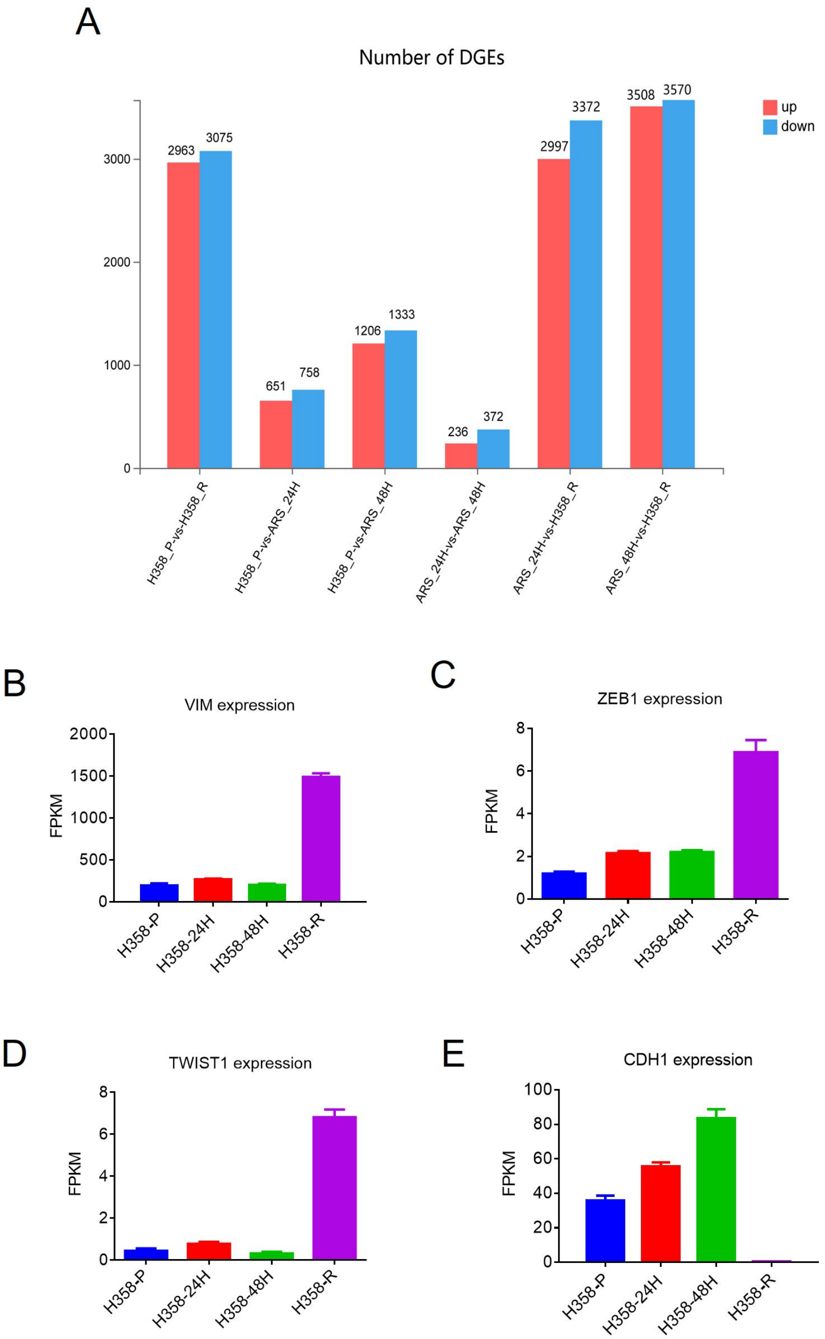


**Figure S1** Transcriptional profiling of H358 cells treated with ARS-1620. (A) RNA-seq was performed with H358_P, H358_24H, H358_48H, and H358_R cells, and the DEGs between the indicated groups. (B-E), The expression of VIM, ZEB1, TWIST1 and CDH1 retrieved from RNA-seq.
