# Supplementary material for: Enhancing the Therapeutic Efficacy of KRASG12C Inhibitors in Lung Adenocarcinoma Cell Models by Cotargeting the MAPK Pathway or HSP90
Source: J Oncol. 2021 Nov 23;2021:2721466. doi: 10.1155/2021/2721466 (PMC8632397; doi:10.1155/2021/2721466)
Supplement: Supplementary Materials — Figure S1: transcriptional profiling of H358 cells treated with ARS-1620. Figure S2: the efficacy of combination therapy with AMG 510 and STA-9090 in vivo. Table S1: information on the antibodies and reagents. [file 2721466.f1.zip › 2721466.f1/Supplemental Table S1.pdf]

Table. S1 Information on the antibodies and reagents.

Categories	Reagents	Manufactures	Catalog numbers	Applications	Notes
Antibodies	ERK	CST, Danvers, MA, USA	9102	WB	1:1000
Antibodies	p-ERK (Thr202/Tyr204)	CST, Danvers, MA, USA	4370	WB	1:1000
Antibodies	S6	CST, Danvers, MA, USA	2217	WB	1:1000
Antibodies	p-S6 (Ser235/236)	CST, Danvers, MA, USA	2211	WB	1:1000
Antibodies	AKT	CST, Danvers, MA, USA	4691	WB	1:1000
Antibodies	p-AKT(Ser473)	CST, Danvers, MA, USA	4060	WB	1:1000
Antibodies	FGFR1	CST, Danvers, MA, USA	9740	WB	1:1000
Antibodies	p-FGFR1 (Tyr653/654)	CST, Danvers, MA, USA	52928	WB	1:1000
Antibodies	Vinculin	Proteintech, Chicago, IL, USA	26520-1-AP	WB	1:1000
Antibodies	Catenin	Thermo Fisher, Waltham, MA, USA	26616	WB	1:1000
Antibodies	HRP-anti-rabbit IgG	Absin, Shanghai, CHN	abs20040	WB	1:10000
Antibodies	HRP-anti-mouse IgG	Absin, Shanghai, CHN	abs20039	WB	1:10000
Antibodies	Ki67	CST, Danvers, MA, USA	12202	WB	1:500
Chemicals	AMG510	Selleck, Houston, TX, USA	S8830	$KRAS^{G12C}$ inhibitor	in 2% HPMC+1% Tween80
Chemicals	ARS-1620	Selleck, Houston, TX, USA	S8707	$KRAS^{G12C}$ inhibitor	in DMSO
Chemicals	AZD4547	Selleck, Houston, TX, USA	S2801	FGFR1 inhibitor	in DMSO
Chemicals	PD0325901	Selleck, Houston, TX, USA	S1036	MEK inhibitor	in DMSO
Chemicals	Ravoxertinib	Selleck, Houston, TX, USA	S7554	ERK inhibitor	in DMSO
Chemicals	GDC-0941	Selleck, Houston, TX, USA	S1065	PI3K inhibitor	in DMSO
Chemicals	STA-9090	Selleck, Houston, TX, USA	S1159	HSP90 inhibitor	in 5% DMSO+45% PEG300
Chemicals	SHP-099	Selleck, Houston, TX, USA	S8278	HSP2 inhibitor	in DMSO
